# Neuromodulation-induced prehabilitation to leverage neuroplasticity before brain tumor surgery: a single-cohort feasibility trial protocol

**DOI:** 10.3389/fneur.2023.1243857

**Published:** 2023-10-02

**Authors:** Leonardo Boccuni, Kilian Abellaneda-Pérez, Jesús Martín-Fernández, David Leno-Colorado, Alba Roca-Ventura, Alba Prats Bisbe, Edgar Antonio Buloz-Osorio, David Bartrés-Faz, Nuria Bargalló, María Cabello-Toscano, José Carlos Pariente, Emma Muñoz-Moreno, Carlo Trompetto, Lucio Marinelli, Gloria Villalba-Martinez, Hugues Duffau, Álvaro Pascual-Leone, Josep María Tormos Muñoz

**Affiliations:** ^1^Institut Guttmann, Institut Universitari de Neurorehabilitació adscrit a la UAB, Badalona, Barcelona, Spain; ^2^Universitat Autònoma de Barcelona, Bellaterra, Barcelona, Spain; ^3^Fundació Institut d'Investigació en Ciències de la Salut Germans Trias i Pujol, Badalona, Barcelona, Spain; ^4^Department of Neurosurgery, Hôpital Gui de Chauliac, Montpellier, France; ^5^Department of Neurosurgery, Hospital Universitario Nuestra Señora de Candelaria, Tenerife, Spain; ^6^Department of Basic Medical Sciences, Universidad de La Laguna, Tenerife, Spain; ^7^Department of Medicine, Faculty of Medicine and Health Sciences, Institute of Neurosciences, University of Barcelona, Barcelona, Spain; ^8^Department of Morphological Sciences (Human Anatomy and Embriology Unit), Faculty of Medicine, Universitat Autònoma de Barcelona, Bellaterra, Barcelona, Spain; ^9^Institut de Recerca Biomèdica August Pi i Sunyer (IDIBAPS), Barcelona, Spain; ^10^Centre de Diagnòstic per la Imatge Clínic, Hospital Clínic de Barcelona, Barcelona, Spain; ^11^Experimental 7T MRI Unit, Magnetic Resonance Imaging Core Facility, Institut d'Investigacions Biomèdiques August Pi i Sunyer (IDIBAPS), Barcelona, Spain; ^12^Department of Neuroscience, Rehabilitation, Ophthalmology, Genetics, Maternal and Child Health, University of Genova, Genova, Italy; ^13^Department of Neuroscience, Division of Neurorehabilitation, IRCCS Ospedale Policlinico San Martino, Genova, Italy; ^14^Department of Neuroscience, Division of Clinical Neurophysiology, IRCCS Ospedale Policlinico San Martino, Genova, Italy; ^15^Department of Neurosurgery, Hospital del Mar, Barcelona, Spain; ^16^Institute of Functional Genomics, University of Montpellier, CNRS, INSERM, Montpellier, France; ^17^Hinda and Arthur Marcus Institute for Aging Research and Deanna and Sidney Wolk Center for Memory Health, Hebrew Senior Life, Boston, MA, United States; ^18^Department of Neurology, Harvard Medical School, Boston, MA, United States; ^19^Centro de Investigación Traslacional San Alberto Magno, Facultad de Medicina y Ciencias de la Salud, Universidad Católica de Valencia San Vicente Mártir, Valencia, Spain

**Keywords:** brain tumor, neuro-oncology, prehabilitation, neuromodulation, neurorehabilitation, neurosurgery, neuroplasticity, clinical trial

## Abstract

**Introduction:**

Neurosurgery for brain tumors needs to find a complex balance between the effective removal of targeted tissue and the preservation of surrounding brain areas. Neuromodulation-induced cortical prehabilitation (NICP) is a promising strategy that combines temporary inhibition of critical areas (virtual lesion) with intensive behavioral training to foster the activation of alternative brain resources. By progressively reducing the functional relevance of targeted areas, the goal is to facilitate resection with reduced risks of neurological sequelae. However, it is still unclear which modality (invasive vs. non-invasive neuromodulation) and volume of therapy (behavioral training) may be optimal in terms of feasibility and efficacy.

**Methods and analysis:**

Patients undertake between 10 and 20 daily sessions consisting of neuromodulation coupled with intensive task training, individualized based on the target site and neurological functions at risk of being compromised. The primary outcome of the proposed pilot, single-cohort trial is to investigate the feasibility and potential effectiveness of a non-invasive NICP protocol on neuroplasticity and post-surgical outcomes. Secondary outcomes investigating longitudinal changes (neuroimaging, neurophysiology, and clinical) are measured pre-NICP, post-NICP, and post-surgery.

**Ethics and dissemination:**

Ethics approval was obtained from the Research Ethical Committee of *Fundació Unió Catalana d'Hospitals* (approval number: CEI 21/65, version 1, 13/07/2021). The results of the study will be submitted to a peer-reviewed journal and presented at scientific congresses.

**Clinical trial registration:**

ClinicalTrials.gov, identifier NCT05844605.

## Introduction

Neurosurgeons performing surgery for brain tumors face a complex dilemma: On the one hand, they must achieve the complete eradication of the tumor; on the other hand, they must preserve the healthy brain tissue surrounding the tumor (1). In fact, radical approaches have the advantage of removing a higher percentage of the tumor, but at the cost of increased risk for post-surgery functional impairments; more conservative approaches have less risks of functional deficits but expose patients to an increased likelihood of developing secondarities. In the last few years, it has been proposed to apply a conditioning intervention before surgery (prehabilitation) to modulate neuroplasticity (2, 3), called neuromodulation-induced cortical prehabilitation (NICP) (4). The objective was to reduce the functional relevance of brain areas close to the tumor (critical areas) in favor of a more distributed brain network, functionally associated with the targeted area but anatomically distant from the tumor. This way, neurosurgeons may apply a more radical approach without the associated risk of functional impairments; from this perspective, it is argued that NICP could represent the optimal therapeutic intervention before intraoperative cortical–subcortical mapping to tailor the resection up to the functional boundaries (hopefully widened by previous neuroplastic changes induced with NICP) (3).

Pioneering efforts have been made in the field, with the publication of four articles (three case reports and one case series) where NICP was undertaken by a total of seven patients with brain tumor (2, 5–7). A common element was neuromodulation coupled with behavioral training: Neuromodulation provokes temporary inhibition (virtual lesion) of eloquent areas, while behavioral training (cognitive/speech or motor training) promotes the activation of alternative brain resources. By performing several sessions of intensive neuromodulation and task-specific training, long-term depression and long-term potentiation mechanisms determine the consolidation of neuroplastic changes before surgery (4, 8).

Notably, NICP by means of *non-invasive* neuromodulation was applied in only one patient, the first case report of NICP published by Barcia et al. (5). The patient (a 59-year-old woman) presented with dysnomia and was diagnosed with left-sided precentral oligodendroglioma (WHO II); during the first brain surgery, the tumor could not be completely removed because of the presence of active language areas. Nine months later, symptoms worsened because of tumor progression. Therefore, before a second surgery, the patient received 13 daily sessions of NICP, consisting of continuous repetitive transcranial magnetic stimulation (rTMS) over Broca's area immediately followed by 10 min of intensive speech training. MEG showed greater bilateralization during speech production, while fMRI with a similar paradigm did not show any change. Language function was temporarily affected after each rTMS session and improved over basal values after each speech training session; along the experiment language function improved, with rTMS having a progressively lower impact. Transient language deficits were present after the second surgery, recovered after 3 weeks, but did not achieve preoperative scores. Surgery was not performed with radical intent, given that an intraoperative biopsy indicated radiation necrosis. Afterward, the patient developed secondarities and died 3 months later.

Since this first case report, it has been inferred that NICP could promote neuroplastic and behavioral changes, but also that higher dosages or different modalities of intervention may have been required (5). In line with these hypotheses, subsequent studies applied radically different NICP protocols, where *invasive* neuromodulation (extraoperative direct cortical stimulation) was applied continuously (24 h a day) at maximal tolerable intensity, with the goal of inhibiting eloquent areas within or near the tumor. In a case series of five patients, Rivera et al. (2) performed first brain surgery, followed by NICP over 15–25 days of therapy, and finally a second surgery (2). During the first surgery, neurosurgeons removed as much tumor mass as possible based on results from cortical and subcortical intraoperative stimulation mapping, while at the same time implanting a grid of subdural electrodes for intracranial electrical stimulation. The subsequent NICP protocol consisted of continuous intracranial stimulation over eloquent areas to inhibit their functionality (virtual lesion), while at the same time providing intensive behavioral training (several hours a day) to patients, to foster the activation of associated functional networks. After several weeks of prehabilitation, a second surgery was performed, where neurosurgeons removed the grid of electrodes and sought to eradicate an additional tumor mass. Results were promising: there was a marked reduction in the functional relevance of eloquent areas, so that during the second surgery, neurosurgeons could remove an additional percentage of tumor mass, without any permanent deficit. However, the main limitations were the need to perform two surgeries and the relatively high rate of adverse events (focal seizure, osteomyelitis, epidural abscess, intermittent myoclonus, and subdural hematoma) caused by surgery and by the presence of intracranial electrodes. More recently, Serrano-Castro et al. (7) published a case report of a 17-year-old patient with a neuroepithelial dysembryoblastic tumor in the left temporo-parietal region provoking refractory focal motor seizures (7), who undertook a similar protocol as described by Rivera-Rivera et al. (2) (first surgery, placement of intracranial electrodes for invasive neuromodulation during subsequent prehabilitation, and second definitive surgery to remove the tumor and the electrodes). However, the first craniotomy was intended specifically for placing electrodes, and NICP was performed for only 6 days, during which language training was provided very intensively (6 h a day). The outcomes were the development of functional activation in the homologous right Wernicke's area and no residual motor language function over the tumor region. Such neuroplastic changes made possible to perform complete tumor eradication, and the patient has since then (at least 1 year) been seizure-free, with no neurological symptoms.

To summarize, seminal results from previous studies indicate that NICP is capable of clinically meaningful neuroplastic changes, although the optimal modality and dosage of therapy remain to be elucidated (4). On the one hand, invasive modalities allow intensive and prolonged inhibition of cortical activity over targeted areas, resulting in meaningful neuroplastic changes, but at the cost of additional surgery and an increased rate of adverse events (2). On the other hand, non-invasive neuromodulation is relatively safe and feasible but has shown less convincing results regarding neuroplasticity (5). However, non-invasive NICP was investigated in only one patient receiving a small, and perhaps insufficient, volume of intervention; further cohort studies exploring different neuromodulation modalities and higher volumes of therapy are needed to determine the feasibility and effectiveness of non-invasive NICP (3, 4).

Therefore, the objectives of the present single-cohort, pilot feasibility trial were to investigate the feasibility (primary outcome) and effectiveness (secondary outcome) of a NICP protocol before brain surgery, consisting of daily sessions of non-invasive neuromodulation over critical areas, followed by intensive behavioral training. We include patients with brain tumors requiring elective neurosurgery. By performing non-invasive neuromodulation within safety guidelines (9), we hypothesize that the protocol is safe and well-tolerated by subjects, with no adverse events and high adherence to the treatment. Second, by comparing neural correlates pre- vs. post-prehabilitation, we will determine whether the intervention is effective in promoting a functional reorganization of the brain, similar to what has already been demonstrated for therapeutic applications of neuromodulation in stroke and other neurological disorders (10). Finally, we will report outcomes post-surgery and the individual's evolution in the long-term recovery phase. The main results will be disseminated by publishing an open-access original research article on the feasibility and effectiveness of non-invasive prehabilitation in neuro-oncology. Furthermore, we will create an online database of case reports with detailed information regarding prehabilitation, surgery, post-surgery rehabilitation, and the long-term evolution of each patient to inform the international community of neurosurgeons and other clinicians in the neurological field.

## Methods and analysis

**Figure 1:**
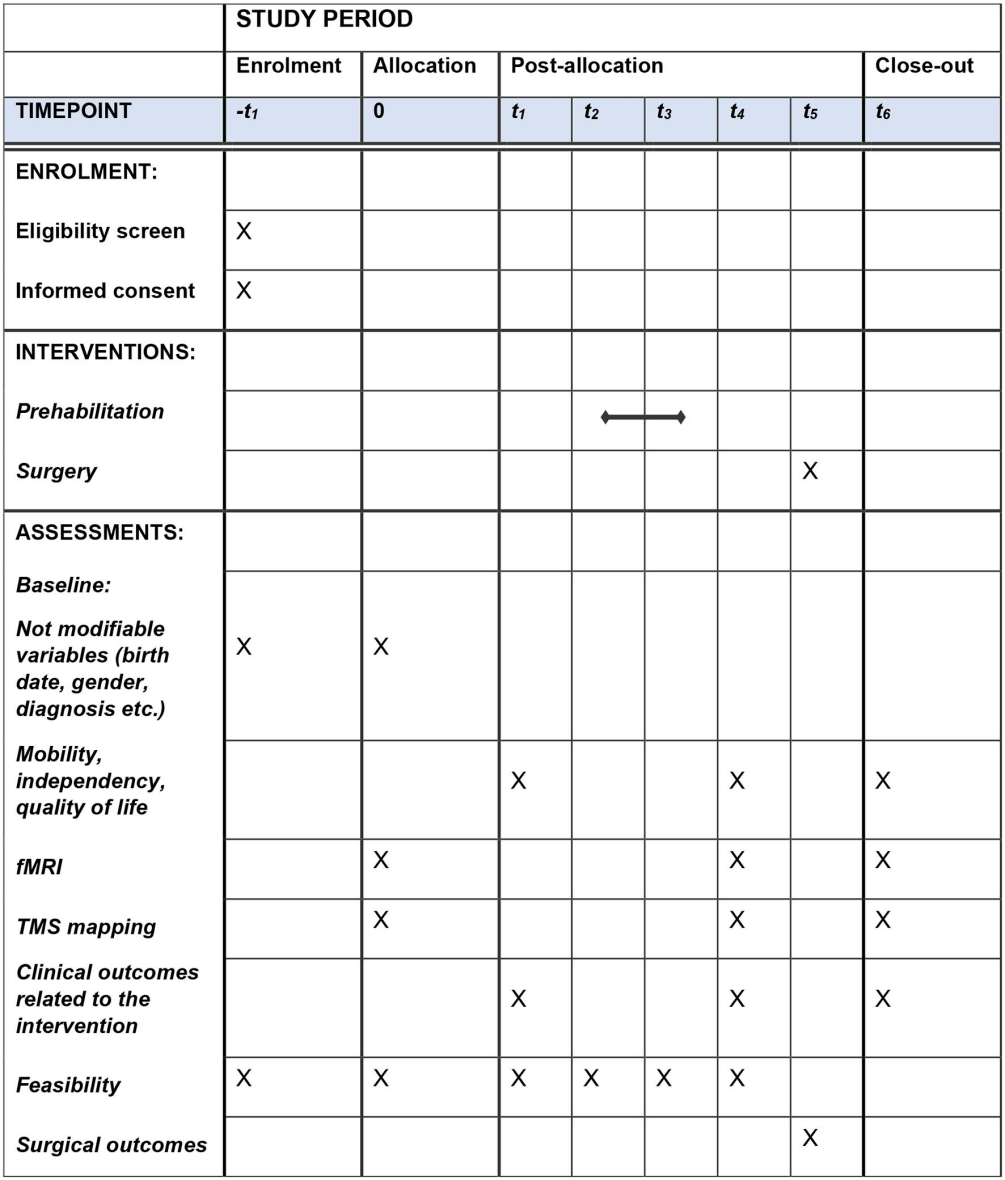
Schedule of enrollment, interventions, and assessments. Interventions at each time point of the study period: -t1 (enrollment): eligibility screen, informed consent, and baseline evaluation. t0 (allocation): fMRI and TMS mapping to determine whether to allocate patients in the prehabilitation program for upper limb or language training; baseline evaluation, if not performed at -t1; assessment of mobility, independency, and quality of life. t1: assessment before starting the prehabilitation protocol; assessment of clinical outcomes related to upper limb or language/cognitive function. t2: first session of prehabilitation. t3: last session of prehabilitation. t4: assessment after the end of the prehabilitation protocol; assessment of clinical outcomes related to the intervention, mobility, independency, quality of life, fMRI, and TMS mapping. t5: surgery. t6: surgical outcomes: intraoperative brain mapping, amount of tumor removed, adverse events, and post-surgery symptoms.

A schematic of the study protocol is outlined in Figure 1. The whole protocol has been developed according to the SPIRIT 2013 guidelines for protocols of clinical trials (11, 12).

### Study settings

Patients on the waiting list for brain surgery are referred by neurosurgeons involved in the study. The principal investigator (JMTM) obtains written informed consent from patients wishing to be enrolled. Participants undertake an articulated, multidisciplinary protocol consisting of clinical, neurophysiological, and neuroimaging assessment, non-invasive brain stimulation, and intensive neurorehabilitation before surgery, neurosurgery, and neurorehabilitation post-surgery. Clinical assessments, neurophysiological investigations, and the whole prehabilitation program are performed at the *Guttmann Institute (Guttmann Barcelona – Brain Health and Neurorehabilitation, Barcelona, Spain)*. Neuroimaging assessment is performed at the *Unitat d'Imatge per Ressonància Magnètica IDIBAPS (Institut d'Investigacions Biomèdiques August Pi i Sunyer) at Hospital Cl*í*nic de Barcelona, Barcelona*. Neurosurgery is performed at the hospitals where the neurosurgeons involved in the study operate. Post-surgery neurorehabilitation is provided at the *Guttmann Institute (Institut Guttmann, Badalona, Spain)*.

### Eligibility

Inclusion criteria are as follows: adults (age ≥ 18 years old) with a diagnosis of brain tumor requiring neurosurgery; ability to undertake at least 10 sessions of the prehabilitation protocol; tumor location posing the patient at risk of developing post-operative neurological deficits, for instance at the level of upper limb motor function and speech production; ability to understand the general purpose of the prehabilitation program and understand simple instructions; being willing to participate and sign the informed consent; being able to sit unassisted for 1 h.

Patients are excluded in cases of any contraindication for magnetic resonance imaging or transcranial magnetic stimulation (9); unstable medical conditions; musculoskeletal disorders that may significantly affect functional training; severe speech and/or cognitive impairment; pain, depression, and fatigue that may significantly affect functional training; and a history of alcohol/drug abuse.

### Primary and secondary outcomes

The primary outcome is the feasibility of the whole intervention, defined by the following parameters:

- Adherence to treatment: to define that the patient completed the protocol, at least 75% of the planned sessions should be performed.
- Retention: successful retention rate is reached if at least 75% of enrolled participants complete the prehabilitation program.
- Adverse events: absence of any adverse event attributable to the prehabilitation program, except for expected transient mild symptoms previously reported for neuromodulation (headache, syncope, and skin irritation) or motor training (mild pain and fatigue).
- Patient's satisfaction: At the end of the protocol, participants fill out questionnaires evaluating the patient's satisfaction with the treatments received (13).

Secondary outcomes are related to exploratory analyses of the effectiveness and potential mechanisms of action of the proposed intervention. We investigate changes from baseline regarding clinical outcomes, fMRI, and TMS mapping. Notably, the goal of the intervention is to reduce the functional relevance of targeted areas because of compensatory activation of other brain resources within the same functional network. Slow-growing tumors already demonstrated that similar neuroplastic changes may occur, with the tumor mass progressively interfering with the functionality of critical areas, while remote brain areas increase their activation; such compensatory mechanisms may explain why these types of tumors are asymptomatic and without any functional deficits in the initial phases (14, 15). From this perspective, the prehabilitation program could be considered a method to artificially optimize and accelerate this neuroplastic adaptation for therapeutic purposes (3). Therefore, we do not expect significant changes regarding clinical outcomes, whereas we consider changes in neuroimaging and neurophysiology outcomes as indicators for the effectiveness of the prehabilitation program.

#### Brain tumor classification and surgical outcomes

For oncological patients, tumor classification is based on the 2021 WHO Classification of Tumors of the Central Nervous System, which represents the most updated taxonomy of brain tumors, and the first classification system considering molecular profiling together with histology (16). Surgical outcomes consider results from intraoperative brain mapping, the absolute and relative amount of tumor removed, adverse events, and neurological status post-surgery.

#### Measurement of motor function, independency, and quality of life

Clinical assessments of upper limb motor function include the following measurements:

- Nine-Hole Peg Test (9HPT) evaluates manual dexterity (17, 18). A dedicated platform is placed in front of the patient, with nine pegs inside a container on the side to be evaluated, and nine holes on the other side. The patient is asked to place pegs into the holes and then put them back in the container, as fast as possible; they are allowed to perform a practice trial before the test trial. The therapist has a stopwatch to measure the time to complete the task and instructs the patient in case of errors (more than one peg picked up at the same time, pegs dropped on the table/floor, etc.). Excellent inter-rater and intra-rater reliability (19, 20) and sensitivity to change have been reported for patients with multiple sclerosis and other neurological disorders (20, 21).
- Fugl–Meyer upper extremity (FM-UE) evaluates upper limb motor impairment, from reflex activity to voluntary motor control out of synergies (22). There are four separate sections, for the assessment of motor function at the level of the arm, the wrist, the hand, and speed–tremor coordination during a finger-to-nose task. Each item is scored as 0, 1, or 2, with a total score ranging from 0 to 66, with lower scores indicating more severe motor impairment. Excellent intra- and inter-rater reliability has been established (23), together with other psychometric measures related to validity, sensitivity, and responsiveness to change (24–26).
- Shoulder abduction finger extension (SAFE) is a quick clinical assessment of upper limb strength, defined as the ability to perform abduction of the shoulder and extension of the index finger; it has potentially high prognostic value for neurological disorders, such as stroke (22). Scoring is usually based on the Medical Research Council scale, ranging from 0 (no visible muscle contraction) to 5 (normal). For the present study, we consider the corresponding items from FM-UE (27).
- To quantify the strength of the hand grip, we use an electronic hand dynamometer. According to the standardized setup, patients are holding the dynamometer while sitting, shoulder adducted and neutrally rotated, elbow at 90-degree flexion, and forearm halfway between pronation and supination (28). They are asked to perform a maximal grip strength effort for 3 s, rest for 60 s, and then repeat the measurement two more times; the average of the three trials is used as the most reliable test result (29). Excellent test–retest reliability has been established (30).
- Reaction time tasks are useful to measure the efficiency of basic processes for perception and response execution. For the present study, we use the Deary–Liewald reaction time task, a freely available program with established validity and reliability (31). In the simple reaction task paradigm, the patient is facing a blue computer screen with one white window in the center, and the index finger of the hand that is being evaluated over the space bar. The instruction is to click on the space bar as soon as an ‘X' appears on the white window; practice trials are allowed before the test trial, which consists of 20 stimuli with a random interval (1–3 s) in between. In the choice reaction task paradigm, there is a similar setup; this time there are four white windows aligned in the center of the screen, and the patient is holding the fingers over the four corresponding letters of the keyboard. Every stimulus consists of an “X” appearing randomly in one of the four windows; patients are instructed to click the corresponding letter as soon as possible. Practice trials are allowed before the formal test trial. For the present study, we adapted the program of reaction time tasks to evaluate one hand at a time without stringent time constraints.

For lower limb motor function, balance, mobility, independency, and quality of life, we consider the following measurements:

- Fugl–Meyer lower extremity: assessment of lower limb motor impairment (22). Each item is scored 0, 1, or 2, with a total score ranging from 0 to 34; low scores are indicative of more severe motor impairment. Excellent reliability has been established for the assessment of motor function after stroke (32).
- Brunel balance assessment: assessment of balance based on 12-item hierarchical tasks, from sitting with arm support to stepping over a step, with excellent reliability and responsiveness to change (33).
- Six-min walking test: submaximal test of aerobic capacity (34, 35). The patient is instructed to walk along a straight path for 6 min, with the goal of covering the longest distance possible within 6 min. Patients are allowed to walk, independently or with the use of assistive devices, and to take rests while standing. Running, sitting, or receiving physical assistance from the therapist (other than help for balance) is not allowed. For the present study, we use a 25-meter path, with visible turning points at the beginning and the end.
- Dual-task assessment is performed, to investigate the interference between cognitive and motor tasks, in particular counting backward three by three during standing balance and gait.
- Neurologic Assessment in Neuro-Oncology (NANO) scale, for the evaluation of neurological functional status in patients with brain tumor (36);
- Karnofsky Performance Status (KPS), for the classification of oncological patients based on the severity of symptoms and their impact on functional independency (37).
- EORTC-QLQ-C30: questionnaire of quality of life for oncological patients (38, 39).
- BN20: EORTC brain cancer module, assessing quality of life specifically for patients with brain tumor (40).
- FA12: EORTC module assessing the impact of fatigue on quality of life for oncological patients (41).

#### Measurement of language/cognitive function

For the clinical evaluation of language and the rest of higher cognitive functions, subtests from the following batteries and neuropsychological tests are used:

- Revised Barcelona Test (TB-R) (42): battery of neuropsychological tests with the aim of assessing high cognitive functions.
- WAIS-III Wechsler Adult Intelligence Scale (43): global intelligence scale for adults that allows for obtaining verbal, manipulative, and total intelligence quotients, as well as indicators of verbal comprehension, perceptual organization, working memory, and processing speed.
- Trail Making Test (44): test used to assess visual attention, sequencing, flexibility, and graphomotor ability. It consists of two parts. In the first part, the subject must place numbers in order along a line; in the second part, the task is to place numbers and letters alternately, in an orderly way.
- Continuous Performance Test-III (CPT-III) (45): computerized test that assesses sustained attention. The subject must press a key whenever a non-target letter appears (if the target is the letter “x,” it should not be pressed).
- Rey Auditory Verbal Learning Test (46): test assessing auditory verbal memory. Fifteen words are read aloud to the subject, who is then asked to repeat as many words as they remember. The procedure is repeated four more times. After some time, a delayed recall of the list is made, and finally, a recognition test is carried out.
- WMS-IV Wechsler Memory Scale (47): scale to assess memory functions.
- Symbol Digit Modalities Test (46): test of attention and visual tracking, concentration, and psychomotor speed. An answer sheet divided into boxes is presented, in which the stimuli are made up of a sequence of geometric figures with a number assigned to each one. The subject must write the number corresponding to each figure in the relevant box, as quickly as possible.
- PMR verbal fluency by letter (48): test assessing lexical access and verbal fluency, in which the subject is asked to say as many words beginning with “P” as they can, within 1 min. The same instructions are given for the letters “M” and “R”.
- Hayling test (49): test that evaluates behavioral regulation, initiation speed, and response inhibition. In the first part, the subject is asked to complete a series of sentences as quickly as possible. In the second part, the subject must complete the sentence with a non-obvious word based on the context.
- Wisconsin Card Sorting Test (50): test evaluating executive function, in particular mental flexibility and abstract reasoning. Four stimulus cards are presented, with different shapes, colors, and number of figures (categories). The subject must match each card in the deck with one of the four key cards (without being told how to do this). The participant only receives feedback on whether the match is correct or incorrect.

Bilingualism is tested by means of a questionnaire (51). Each participant is going to be self-rated on a 4-point scale on the abilities of comprehension, Reading, writing, fluency, and pronunciation for each language (1 = poor, 2 = regular, 3 = good, and 4 = perfect). In addition, a laterality test is included, the Edinburgh Handedness Inventory (52), as well as a questionnaire to assess anxiety and depression, the Hospital Anxiety and Depression Scale (53).

#### Neuroimaging

Magnetic resonance imaging (MRI) is acquired to assess anatomical and functional (fMRI activation maps) brain changes due to intervention and surgery. For instance, the development of novel activation sites distant from the surgical target may indicate that NICP was effective in promoting a reduction of functional relevance for targeted areas, in favor of a more distributed network. Therefore, all patients undergo three identical MRI sessions: (1) before NICP, (2) after NICP but before surgery, and (3) after surgery. Each session consists of MRI data acquisition using a 3 Tesla Siemens PRISMA scanner and a 32-channel head coil. The protocol includes accelerated multiband sequences adapted from the Human Connectome Project and provided by the Center of Magnetic Resonance Research (CMRR) at the University of Minnesota.

Regarding anatomical acquisitions, a high-resolution T1-weighted structural image is obtained with a magnetization-prepared rapid acquisition gradient-echo (MPRAGE) three-dimensional protocol, in which in an ascending fashion a total of 208 contiguous axial slices are obtained [repetition time (TR) = 2400 ms, echo time (TE) = 2.22 ms, inversion time = 1000 ms, flip angle = 8°, field of view (FOV) = 256 mm and 0.8 mm isotropic voxel]. In the same session, a high-resolution multishell diffusion-weighted MRI scan is obtained. This scan consists of two multiband acquisitions (anterior–posterior; acceleration factor = 4), sensitized in 99 monopolar directions with a b-value of 3000 s/mm2 in an echo-planar imaging sequence [TR = 3230 ms, TE = 89.20 ms, section thickness = 1.5 mm, voxel size = 1.5 × 1.5 × 1.5 mm, FOV = 210 mm].

In terms of quantifying brain function, resting-state and task functional MRI (fMRI) are acquired. Both consist of high-resolution multiband (anterior–posterior phase-encoding, acceleration factor = 8) interleaved acquisitions [T2^*^-weighted EPI scans, TR = 800 ms, TE = 37 ms, 750 volumes, 72 slices, slice thickness = 2 mm, FOV = 208 mm]. First, resting-state fMRI is acquired while the patient is instructed to keep his eyes closed and remain still without falling asleep.

Then, task fMRI is acquired during three language and three motor paradigms, which adhere to the following procedures:

- Word generation task: Block paradigm consisting of five cycles. Each cycle comprises 30 s of rest followed by 30 s during which the participant must mention words starting with a certain letter. These letters are “F”, “A”, “S”, “M”, and “E”, in this same order.
- Semantic decision task: Block paradigm consisting of five cycles. Each cycle comprises 30 s of rest followed by 30 s during which the participant must mention objects from certain places: school, kitchen, car, house, and hospital.
- Comprehensive auditory task: Block paradigm consisting of three cycles. Each cycle comprises a 30 s block, in which a story is narrated in a made-up language (inactive/rest block/condition), followed by another 30 s block, in which a story is narrated in Spanish (active block/condition).
- Finger tapping task: Block paradigm consisting of three cycles. Each cycle comprises a 30 s block, in which the patient is asked to do a fingering exercise (tap each finger with the thumb) in the corresponding hand, followed by another 30 s of rest.
- Ankle flexion task: Block paradigm consisting of three cycles. Each cycle comprises a 30 s block, in which the patient is asked to move the corresponding foot up and down slowly, followed by another 30 s of rest.
- Tongue movement task: Block paradigm consisting of three cycles. Each cycle comprises a 30 s block, in which the patient is asked to move the tongue in circles without opening the mouth, followed by another 30 s of rest.

#### TMS for assessment and modulation of brain function

**Figure 2:**
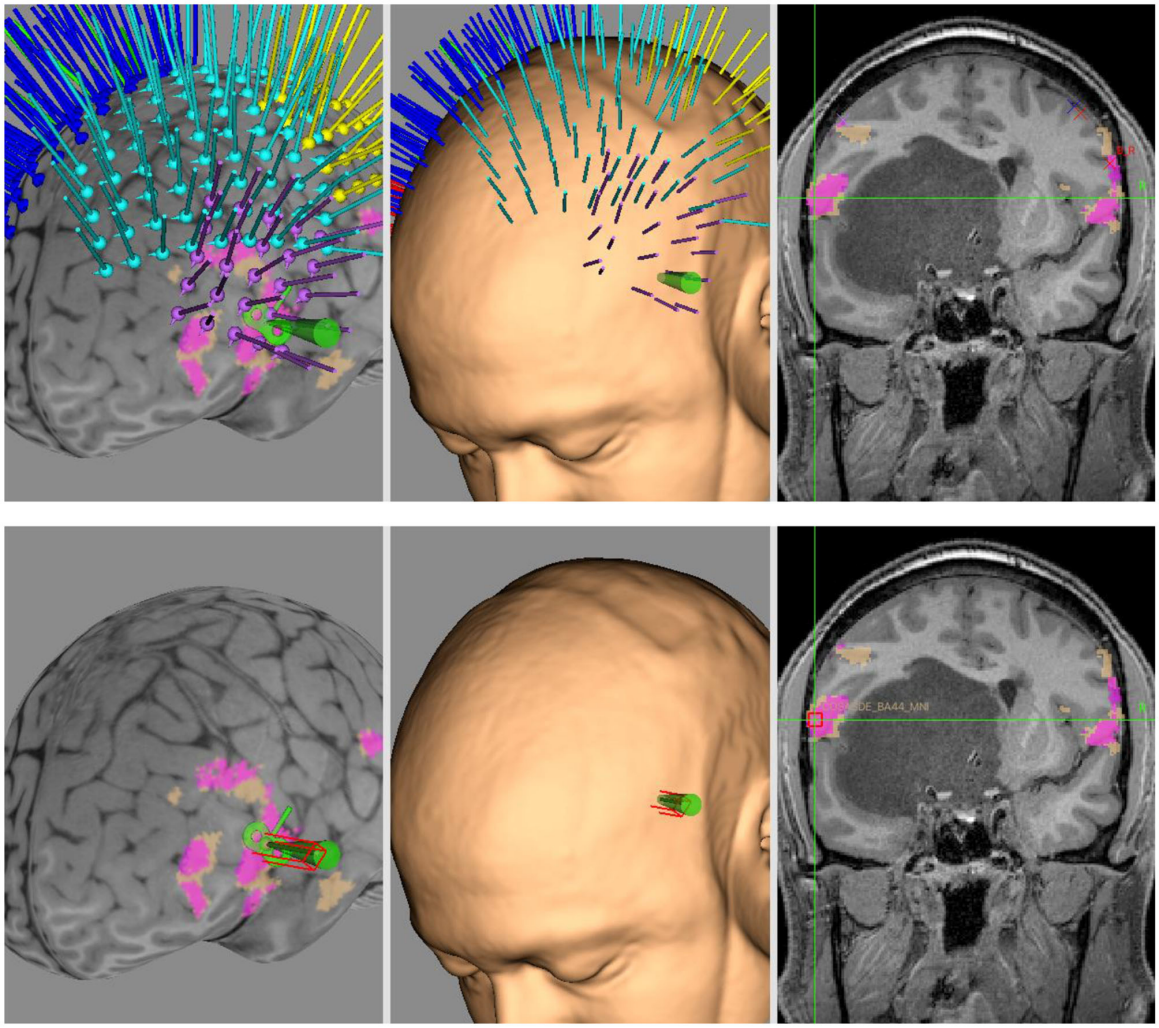
Neuronavigated TMS mapping and neuromodulation. Example of Brainsight project developed for neuronavigated TMS mapping of motor and language functions (upper row) and for subsequent neuromodulation by means of low-frequency rTMS (lower row). In this case, a patient with subcortical frontotemporal tumor undertook NICP for language function, with the target of stimulation based on MNI coordinates of peak fMRI activity for ipsilesional semantic decision task. The overlays in fuchsia and beige are clusters of fMRI for word generation task and semantic decision task, respectively.

Neuromodulation by means of TMS is used for assessment and therapeutic purposes, to both non-invasively estimate the excitability and modulate the plasticity of the cerebral cortex (Figure 2).

Typical assessments are single-pulse and paired-pulse protocols (54, 55). With single-pulse, it is possible to determine the resting motor threshold (RMT) and then measure contralateral peripheral response to suprathreshold motor evoked potentials (MEPs) during motor mapping of the upper limb, lower limb, and facial muscles. When a suprathreshold MEP is delivered over contralateral M1 during an isotonic muscle contraction, a cortical silent period (transient disruption of EMG activity) is visible immediately after the stimulus and is a measure of intracortical inhibitory circuitry. Other assessments of intracortical inhibition are investigated by paired-pulse protocols, such as short-interval and long-interval paradigms where the inter-stimulus interval is between 1–5 ms and 50–200 ms, respectively. By contrast, paired-pulse at intervals between 8 and 30 ms cause intracortical facilitation.

There are several paradigms of repeated TMS (rTMS) to promote neuroplastic changes (9, 56). Conventional rTMS has inhibitory effect at low frequency ( ≤ 1 Hertz) and excitatory effect at high frequency (>1 Hertz). For patterned (theta-burst stimulation, TBS) rTMS, inhibitory and excitatory effects result from continuous TBS and intermittent TBS, respectively. Stimulation parameters (stimulation intensity, number of pulses) and external factors (medications, drugs, mental status) may significantly alter or even reverse the effect of the neuromodulation (57, 58); therefore, treatment sessions should be performed in standard conditions and following specific protocol parameters.

#### Neurophysiological assessment

By performing neuronavigated TMS mapping, we compare the anatomical distribution of active targets pre- vs. post-intervention. The same targets and intensity of stimulation defined at baseline are used at the end of the intervention, to allow comparisons. A figure of 8 coil (MagPro Cool B65-AP-RO Coil) connected to a transcranial magnetic stimulator (MagVenture MagPro x100) is driven by a robotic arm (Axilum Robotics TMS-Cobot) controlled manually or through a dedicated neuronavigation software (Brainsight TMS neuronavigation). We apply biphasic current with an anterior–posterior followed by a posterior–anterior current direction in the brain; the TMS coil is held tangential to the scalp, the handle pointing backward with a 45-degree deviation from the sagittal plane. For curvilinear 3D brain reconstruction (MNI coordinates), participant's structural and functional MRI data are uploaded. Grids of targets (inter-target distance: 10 mm) are defined based on anatomical landmarks for the subsequent motor/language mapping protocol and to determine the hotspot for neuromodulation. Grids are placed in correspondence with the primary sensorimotor and premotor areas (motor mapping) and over the pars triangularis and the gyrus supramarginalis (language mapping).

The procedure for motor and language mapping (at baseline and at the end of the prehabilitation program) is as follows:

*Set up*. The patient is seated on an electromechanics treatment chair to ensure comfort and stability during the assessment. An optic tracker (Polaris Vicra) detects trackers for 3D localization of the Cobot, the patient (a Brainsight Adhesive Subject Tracker is attached to the patient's forehead), and the pointer (Brainsight P-970, for the registration of anatomical landmarks). After skin preparation (alcohol swab), self-adhesive electrodes for EMG recording are attached bilaterally to the olecranon (ground), the muscle belly of the first dorsal interosseus (FDI, negative electrode), and the muscle tendon of the FDI (positive electrode). A pillow is placed underneath the forearm of the side being assessed, to ensure that muscles are completely at rest during the protocol. PowerLab 8/35 and Quad Bio Amp (ADInstruments, data acquisition hardware devices) register the EMG response and send data to the dedicated software (ADInstruments, LabChart, data analysis software). Every time a TMS pulse is delivered, a trigger signal is sent automatically from the MagPro x100 to the PowerLab through a D-type 26-BNC interface cable, to initiate the recording of the EMG response. For language mapping, a screen for image presentation is placed in front of the patient. One computer runs Brainsight, while another computer runs LabChart and a dedicated MATLAB script for language mapping.

*Mapping protocol*. The whole protocol is performed for both the affected and unaffected sides. For motor mapping, the entire session is recorded in LabChart. A preliminary search of the hotspot is performed around the hand knob of the precentral gyrus, starting from an intensity of 35–40% and rising progressively up the intensity until stable MEPs (here defined as signals with peak-to-peak amplitude larger than 500 μV) are produced. Then, a formal hotspot search is performed, by applying five stimuli to each selected target; the target showing the largest average MEPs is considered the hotspot to determine the RMT, defined as the lowest intensity capable of eliciting three MEPs (signals with peak-to-peak amplitude larger than 50 μV) out of six consecutive stimuli (5- to 10-s interval). Once RMT has been determined, motor mapping is performed by applying five stimuli for each target (120% RMT intensity, 5- to 10-s interval) as far as positive MEPs (signals with peak-to-peak amplitude larger than 50 μV) are detectable.

For language mapping, a speech disruption protocol is applied. Initially, patients are familiarized with pictures and instructed to name them as soon as they appear on a screen in front of them. Once it is verified that the patient can name pictures correctly, we start language mapping. rTMS (five pulses at 5 Hertz, 90% RMT) is delivered to each target, together with presenting a picture on the screen (picture presentation time: 500 ms; delay picture-rTMS: 0 ms). The order of target stimulations and picture presentations is pseudo-randomized. An audio/video recording of the patient's response (from 0 to 3000 ms after the first rTMS stimulus) is evaluated offline by a neuropsychologist unaware of the target that has been stimulated to determine whether there was an episode of speech arrest, anomia, or other speech disruption phenomena. The language protocol is repeated until each target receives rTMS three times. A dedicated MATLAB script is developed to automatically synchronize rTMS with picture presentation and audio–video recording.

### Interventions

Patients are scheduled for a minimum of 10 and a maximum of 20 treatment sessions, distributed as one/two sessions each weekday. Each session consists of 30 min of neuromodulation (rTMS or tDCS) coupled with 60 min of intensive motor or language training. The decision on whether to provide rTMS or tDCS is individualized based on specific patient and lesion characteristics.

Notably, the coupling between neuromodulation and task training represents a therapeutic application of the concept of metaplasticity. In fact, metaplasticity has been defined as any change in the direction or degree of synaptic plasticity based on prior neural activity (59). A typical study design investigating metaplasticity applies multiple rTMS sessions within the same day (accelerated rTMS), with minutes/hours between sessions (60). Compared to single rTMS, it has been demonstrated that accelerated rTMS may produce additive strengthening of neuroplastic changes, both for excitatory (60) and inhibitory (61) paradigms. Another way of exploiting the therapeutic potential of metaplasticity is through the combination of rTMS with task training. Previous systematic reviews and randomized controlled trials have already demonstrated the beneficial effect of rTMS delivered before therapy for aphasia, lower limb, and upper limb motor function in stroke patients (62–64). In this perspective, for the present study, neuromodulation is the metaplastic ‘primer' setting the direction and degree for subsequent neuroplastic changes promoted by intensive task training.

#### Neuromodulation – Navigated rTMS

rTMS is applied with the following parameters: waveform: monophasic; intensity: 90% resting motor threshold (RMT); pulse frequency: 1 Hertz; total number of pulses: 1800. The choice of the target is determined based on anatomical considerations and results from neurophysiological and neuroimaging assessments; hence, the area of highest activity among those structures anatomically located near the tumor is considered the target. For instance, by performing motor and language mapping, we could identify active targets at the level of targeted areas, such as the hand knob or sites where speech arrest was detected. We also consider the center of mass of fMRI mapping related to hand movement and speech production as potential hotspots for neuromodulation.

#### Neuromodulation – tDCS

In those cases of subcortical tumors potentially affecting a wider area and more than one function, tDCS is performed with the aim of inhibiting (cathodal stimulation) distributed areas related to functions potentially at risk of being compromised, while at the same time promoting the activation (anodal stimulation) of safer areas within the same functional network.

#### Training of functions at risk of being compromised

Training is performed immediately after inducing a virtual lesion, focusing on functions related to structures targeted by neuromodulation. Motor, language, and/or cognitive functions are selected at the beginning of the protocol, considering the anatomical location of the tumor, medical condition, and pattern of activation by each of the assessed functions in the fMRI.

Detailed design and monitoring of training sessions are gathered, including goals pursued in any session, assigned activity, number of repetitions, and performance.

##### Prehabilitation motor training

Patients undertake intensive motor training sessions soon after (TMS) or during (tDCS) neuromodulation, i.e., in the condition of a virtual brain lesion (targeted area temporarily inhibited). This way, the brain must recruit alternative resources, which cumulatively determine neuroplastic changes and a stable shift of functional activation patterns away from critical areas.

There is a potentially infinite range of activities that could be trained; to be systematic, we defined a taxonomy of exercises based on anatomical and functional domains. Considering, for instance, the upper limb, we decided to prioritize tasks based on proximal (reaching and hand orientation) or distal (finger individuation and manipulation) components of upper limb movements. Within each component, we consider the integration between upper limb motor function and other body segments and/or task domains. For instance, some tasks may be accomplished by using one arm or hand (unimanual), while others may require the cooperation of both limbs (bimanual coordination) or additional balance and/or cognitive challenges (sequence learning, motor memory, and dual task). The goal is to cover all aspects of upper limb functionality and possibly to promote network connectivity of cortical–subcortical–cerebellar structures related to motor control and executive function, beyond the predominant activation of motor–premotor areas of the affected side (65–68).

Other important concepts of motor learning that we consider are task difficulty, task intensity, and task variability (69). Task difficulty refers to the type of challenge that we impose; for instance, speed, accuracy, the ability to isolate or integrate different motor, and/or cognitive tasks. Task intensity refers to the necessity of a high number of repetitions to promote neuroplastic changes associated with motor learning. Task variability is necessary to avoid patient boredom and generalize the benefits of the training; in fact, training always on the same task makes you proficient specifically on that task, while varying training conditions foster retention and generalization of learning to new tasks (70).

Finally, we should point out that most patients are relatively young adults with limited to no symptoms of motor/cognitive impairments before surgery. Therefore, we consider challenging activities that resemble sports and playful games, such as hitting targets, playing ball games, playing the Piano, and manipulating objects. Two common elements of all interventions are (1) that task performance is made up of discrete repetitions and (2) that outcomes are quantitatively measurable. For instance, preparing a meal, dressing, or tidying the table are motor tasks that cannot be easily divided into repetitions or whose outcome is quantitatively defined. By contrast, hitting targets or playing the piano are made of discrete individual repetitions, require specific spatial and temporal accuracy, the success/error rate can be easily measured, and progression can be monitored over time. To summarize, we prioritize goal-oriented, challenging tasks for their positive impact on neuroplasticity (network activation and connectivity), motor learning (difficulty, intensity, and variability), and motivation (constant monitoring of performance).

Virtual reality is also used to promote the activation of non-canonical pathways related to function (71). Any user can effectively distinguish immersive virtual reality from the real world, evidencing different enough recruited networks. However, training in a virtual environment transfers to real-environment learning (72, 73). This well-documented phenomenon enables virtual reality training to promote the recruitment of alternative pathways in the context of preferential ones.

##### Prehabilitation language and cognitive training

Similar to motor training, sessions are performed soon after TMS or during concomitant tDCS. Patients undertake computerized cognitive training sessions on the online rehabilitation platform “Guttmann NeuroPersonalTrainer”^®^ (GNPT) (74), with a duration of ~60 min per session. Tasks aim at language training (60% of the tasks) and other cognitive functions (40%), consisting of a set of personalized cognitive exercises based on the initial neuropsychological assessment that allows for establishing the profile of cognitive impairment. These tasks are adequately parameterized. To this end, neuropsychologists may define a set of input parameters for every task, such as presentation speed, latency time, or number of images, allowing personalization based on different difficulty levels. Regarding language, tasks are planned and supervised in a personalized way by a neuropsychologist, readjusting their planning if necessary.

Language tasks are oriented to naming and generating words, although other tasks oriented to language expression (grammar, semantics, and writing) and language comprehension (reading, comprehension of words, sentences, texts, and listening) could be contemplated.

For the rest of the cognitive functions, GNPT platform allows programming the following functions: (1) temporospatial orientation; (2) attention (selective, sustained, and divided); (3) memory (visual, verbal, and working memory); (4) executive functions (planning, inhibition, flexibility, sequencing, and categorization); (5) visual gnosis; (6) mental calculation; and (7) constructive praxis.

Furthermore, cognitive training is complemented by the telerehabilitation platform of the Barcelona Brain Health Initiative (BBHI) (75).

Finally, we apply specific tasks to train bilingualism. The objective is to achieve the disturbance of linguistic tasks through the temporary inhibition of the critical areas near the lesion (peritumoral), so that the brain can find alternative resources and facilitate neuroplasticity processes. This linguistic disturbance is made according to the representation of the tumor area (areas of higher functional compromise), to later realize language tasks, such as naming, comprehension, and fluency tasks in two languages (Spanish/Catalan), to enhance residual activities and promote a reorganization of functions during the disturbance. Prior to these procedures, a bilingualism questionnaire is administered to assess the dominant language of each participant.

### Discontinuation, adherence, permission for concomitant care, ancillary, and post-trial care

Intervention is discontinued at the participant's request or in case of adverse events attributable to neuromodulation (seizure). Adherence to treatment is monitored by recording the rate of sessions attended over the total number of planned sessions.

Patients are allowed to undertake any concomitant care during the intervention if it does not interfere with the schedule of the prehabilitation program. However, we also recommend patients avoid other neuromodulation or motor skill training approaches, as they may be counterproductive to the desired outcome of the intervention. For instance, undertaking additional upper limb training sessions outside the prehabilitation protocol may reinforce the activation of peritumoral areas, as the inhibitory effect of neuromodulation might not be present anymore.

In case of post-surgery cognitive and/or neurological deficits, neurorehabilitation (usually 10 to 30 sessions) will be provided by the Guttmann Institute as a service offered to patients who have participated in the research study.

### Dissemination

A summarized version of the study protocol has been published online with ClinicalTrials.gov Identifier NCT05844605 (more information of the SPIRIT WHO trial registration data set are reported in Table 1).

**Table 1:** SPIRIT WHO trial registration data set.

**Data category**	**Information**
Primary registry and trial identifying number	ClinicalTrials.gov (NCT05844605)
Date of registration in primary registry	04/05/2023
Secondary identifying numbers	Protocol ID 2020330
Source(s) of monetary or material support	Fundación Joan Ribas Araquistain (reference project 2020.330) Fundaciò La Maratò De TV3 (reference project 201735.10) Fundaciò Bancària La Caixa (reference project LCF/PR/PR16/11110004)
Primary sponsor	Institut Guttmann, Institut Universitari de Neurorehabilitació adscrit a la UAB, Badalona, Spain
Contact for public or scientific queries	Name: José María Tormos Muñoz Email address: jmtormos@guttmann.com Telephone: 934 977 700 Postal address: Camí de Can Ruti, s/n 08916 Badalona, Spain
Title	Neuromodulation-Induced Prehabilitation to leverage neuroplasticity before surgery for brain tumors: protocol for a single-cohort feasibility trial.
Countries of recruitment	Spain
Health condition(s) or problem(s) studied	Primary brain tumor
Intervention(s)	Neuromodulation, behavioral training, neurosurgery
Key inclusion and exclusion criteria	Inclusion: diagnosis of primary brain tumor requiring neurosurgery Exclusion: contraindications to TMS or MRI
Study type	Single-arm pilot feasibility trial
Date of first enrollment	21 June 2021
Target sample size	20
Recruitment status	Recruiting
Primary outcome(s)	Feasibility
Key secondary outcomes	Changes pre-/post-intervention on clinical, neurophysiology, and neuroimaging outcomes

Study data are collected and managed using REDCap electronic data capture tools hosted at Guttmann Institute (76, 77). To ensure confidentiality, each study participant is identified with an alphanumerical code. Data from neurophysiology and neuroimaging are anonymized and stored on online cloud platforms. Accessibility to data files is granted by the principal investigator only to researchers involved in data management. The hard copy of informed consent forms and other data collected on paper is stored in a locked closet at the Guttmann Institute, accessible only by the principal investigator.

Anonymized data supporting study findings are available from the corresponding author, upon reasonable request. Alternatively, an online data repository named “Joan Ribas Araquistain Dataset on Brain Tumor Prehabilitation” is created and made accessible upon reasonable request to accredited clinicians, researchers, and institutions in the field of neuro-oncology. Furthermore, we sought to establish collaboration agreements with international oncology databases, such as the Georgetown Database of Cancer (G-DOC), REMBRANDT (Repository of Molecular Brain Neoplasia Data), and the Cancer Imaging Archive.

The results of the study will be submitted to a peer-reviewed journal and presented at scientific congresses.

### Statistical analysis

We use R software for statistical analysis and graphics (78). Given the small sample size (20 patients, based on a realistic estimate of the recruitment rate) and the use of ordinal scales, we perform non-parametric statistics. For descriptive reporting of continuous/ordinal variables, median and interquartile range (IQR) are used to indicate measures of central tendency and dispersion, respectively; frequencies are reported by indicating the absolute value, followed by the relative value (percentage) in brackets; in case of binary variables (such as gender), only one of the two variables is reported. For the primary analysis (feasibility), we report descriptively whether we met the criteria for recruitment, retention, and adverse events; adherence to treatment (both neuromodulation and behavioral training) is reported as the median (IQR). An exploratory analysis of effectiveness is conducted by performing a repeated-measure comparison (pre- vs. post-prehabilitation). Together with reporting the estimates of treatment effect, we indicate the actual level of significance (two-sided *p*-value) and 95% confidence interval (79). For quantitative and ordinal variables, we use Wilcoxon signed-rank test. For dichotomous variables, we use McNemar's test. For correlations between quantitative/ordinal variables measured at the same time point, we use Kendall's tau rank correlation coefficient. To explore prediction models, we use simple and multiple linear regression analyses for continuous/ordinal outcomes and simple and multiple logistic regression for binary outcomes. In case of missing data, we perform pairwise deletion. Further explorative analysis may include big data analysis of neuroimaging/neurophysiology data.

## Ethics statement

All procedures from the present study were performed in accordance with the Helsinki Declaration. Ethics approval was obtained for the present experimental protocol by the Research Ethical Committee of Fundació Unió Catalana d'Hospitals (approval number: CEI 21/65, version 1, 13/07/2021). Patients provide written informed consent before being enrolled in the study.

## Author contributions

LB, ÁP-L, and JT conceived the manuscript. LB, DB-F, NB, and MC-T initiated the study design. KA-P, JM-F, DL-C, JP, and EM-M helped with implementation. All authors contributed to the refinement of the study protocol and approved the final manuscript.

## Neuromodulation-induced prehabilitation in the brain tumour surgery group

Andreu Gabarrós Canals (Hospital de Bellvitge), Alberto Di Somma, Joaquim Enseñat, Jordi Rumià Arboix, José Poblete Carrizo, and Ramón Torné Torné (Hospital Clínic de Barcelona), Carlos Domínguez Alonso, Ferran Brugada Bellsolà, and Pilar Teixidor Rodríguez (Hospital Germans Trias i Pujol), Cristóbal Perla y Perla, Estela Lladó Carbó, Mireia Illueca Moreno (Hospital HM Nou Delfos), Cristian de Quintana, Fernando Muñoz Hernández, and Gloria Villalba Martinez [Hospital del Mar y Hospital de la Santa Creu i Sant Pau (Servicio de Neurocirugía mancomunado)], Mònica Buxeda Rodríguez (Hospital Mutúa de Tarrasa), Fabián Romero Chala (Hospital Parc Taulí), Fran Martinez Ricarte (Hospital Vall d'Hebron).
